# Increased serum human epididymis protein 4 is associated with disease activity and systemic involvement in pediatric-onset systemic lupus erythematosus

**DOI:** 10.3389/fimmu.2024.1461987

**Published:** 2024-09-10

**Authors:** Chenxi Liu, Lingyi Yan, Menglan Zhang, Yifei Duan, Jie Luo, Zhijun Liu, Ting Liu, Jiyu Tong, Yongmei Jiang

**Affiliations:** ^1^ Department of Laboratory Medicine, West China Second University Hospital, Sichuan University, Chengdu, Sichuan, China; ^2^ Key Laboratory of Birth Defects and Related Diseases of Women and Children, Ministry of Education, Sichuan University, Chengdu, Sichuan, China; ^3^ West China Second University Hospital, Sichuan University, Chengdu, China; ^4^ Department of Immunology, West China School of Basic Medical Sciences and Forensic Medicine, Sichuan University, Chengdu, China

**Keywords:** correlation, human epididymis protein 4, lupus nephritis, pediatric-onset systemic lupus erythematosus, biomarker

## Abstract

**Objective:**

We aimed to investigate human epididymis protein 4 (HE4) as a potential biomarker in patients with pediatric-onset systemic lupus erythematosus (pSLE), particularly on the association of serum HE4 levels with disease activity and other laboratory tests.

**Methods:**

We included 137 patients with pSLE and 75 age- and sex-matched healthy controls (HCs). Serum HE4 level was measured by a chemiluminescent microparticle on an Abbott ARCHITECT i2000SR Immunoassay Analyzer. Comparisons between groups were performed using the independent Student t-test, Mann–Whitney U test, Chi-square test, or Fisher’s exact test, as appropriate. We also determined the relationships between HE4 and clinical parameters and evaluated disease activity using SLE Disease Activity Index (SLEDAI) and renal SLEDAI (rSLEDAI).

**Results:**

Serum HE4 levels in patients with pSLE (44.6 pmol/L; IQR, 32.5–73.5) were significantly higher than those in HCs (38.9 pmol/L; IQR, 34–46.1). HE4 levels were significantly higher in moderate to severe disease activities (57.4 pmol/L, IQR 37.7–164.5) than in mild disease activities (38.8 pmol/L, IQR 30.1–48.5) or HCs (38.9 pmol/L, IQR 34.0–46.1), as well as in active renal disease activities (77.2 pmol/L, IQR 47.4–224.1) than in inactive renal disease activities (36.1 pmol/L, IQR 27.8–46.7). The ROC curve analysis showed that HE4 could discriminate pSLE with renal (AUC, 0.717; 95% CI, 0.632–0.801), hematological (AUC, 0.740; 95% CI, 0.648–0.831), and cardiovascular involvement (AUC:0.775, 95% CI 0.669–0.880). Serum HE4 levels significantly correlated with several indicators related to renal morbidity, such as creatinine, blood urea nitrogen, uric acid, cystatin C, urine protein/24 h, etc.

**Conclusion:**

Serum HE4 levels in pSLE were elevated and highly associated with disease activity and systemic involvement, indicating HE4 as a potential biomarker for pSLE.

## 1 Introduction

Pediatric-onset systemic lupus erythematosus (pSLE) is a complex and chronic autoimmune disease primarily affecting children and adolescents, characterized by disease onset at age <18 years (1). pSLE presents unique challenges compared with adult-onset systemic lupus erythematosus (SLE) due to its distinct clinical manifestations, disease course, and impact on growth and development (2–4). Symptoms of pSLE can range from mild to severe. Common manifestations include joint pain, skin rashes, fever, fatigue, and organ involvement, such as the kidney, brain, heart, and lung (5–7). Diagnosing pSLE requires careful evaluation of clinical symptoms, laboratory tests, and imaging studies. The American College of Rheumatology and the Systemic Lupus International Collaborating Clinics have established the criteria for SLE diagnosis, including the presence of specific clinical features and the detection of antinuclear antibodies in the blood (8). Laboratory assessment helps establish pSLE diagnosis, monitor disease activity, and detect disease exacerbations, indicating that laboratory examinations are crucial in SLE diagnosis (3). The systemic lupus erythematosus disease activity index (SLEDAI) score is widely utilized to measure lupus disease activity, offering numerical scores derived from a comprehensive assessment of laboratory and clinical symptoms. Due to the higher SLEDAI scores indicating increased risk of organ damage and mortality risk, regular monitoring of disease activity and organ damage is necessary for patients with pSLE (9).

Human epididymis protein 4 (HE4), also known as whey acidic protein four-disulfide core domain protein 2, is a glycoprotein initially identified as a protein predominantly expressed in the epididymis (10). Recently, it has gained significant attention as a biomarker for specific gynecological conditions, particularly ovarian (11, 12) and endometrial carcinomas (13, 14). Given the increasing risk of cancer among patients with autoimmune diseases (15), tumor biomarkers including HE4 are currently being employed to assess cancer risk. It has been investigated as a potential biomarker for autoimmune diseases, including SLE, rheumatoid arthritis (RA) (16), primary Sjögren syndrome (pSS) (17), idiopathic inflammatory myopathies (IIM) (18), and IgG4-related disease (IgG4-RD) (19). Recently, HE4 has been recognized as a biomarker for fibrotic disease and is involved in the pathology of fibrotic diseases (20). Recent studies have found that HE4 was significantly elevated in connective tissue disease-related pulmonary or renal involvement (21–23).

Based on these observations, our study sought to delve into the serum HE4 levels among patients with pSLE. Although several studies have shown that serum HE4 levels are elevated in patients with adult-onset SLE, particularly in those with lupus nephritis (LN) (24), its relationship with disease activity in pSLE remains unclear. The assessment of disease activity in SLE continues to pose significant challenges both in clinical practice and research settings. Therefore, we aimed to investigate the serum HE4 levels in patients with pSLE and its association with disease activity, clinical characteristics, and other laboratory tests for potential clinical applications in this study.

## 2 Materials and methods

### 2.1 Study population

We conducted this study at the West China Second University Hospital of Sichuan University between January 2022 and December 2023. We enrolled 137 patients with pSLE and 75 age-and sex-matched healthy controls (HCs). All patients met the 2021 Chinese Guidelines for the Diagnosis and Treatment of Childhood-onset Systemic Lupus Erythematosus for pSLE (25). The SLEDAI scoring system in the Chinese guideline was used to calculate disease activity scores: mild, 0–6; moderate, 7–12; severe, ≥13. In addition, renal involvement was assessed using renal SLEDAI (rSLEDAI), consisting of four renal-related items of SLEDAI (26), such as hematuria (>5 red blood cells [RBCs]/high-power field [HPF]), pyuria (>5 white blood cells/HPF), proteinuria (>0.5 g/24 h or urine protein/creatinine ratio of >0.5), and urinary casts (heme, granular, or red blood cells), with each scored 4 points. Patients with rSLEDAI score of ≥4 were considered to have active renal disease activity. Disease duration was defined as the time from symptoms to diagnosis. The Ethics Committee of West China Second University Hospital of Sichuan University approved this study (Protocol number: 2023-289).

### 2.2 Sample collections and HE4 measurement

Serum remnants from blood samples retrieved for routine laboratory tests were collected and stored at −80°C until HE4 measurements. Serum HE4 levels were measured using a chemiluminescent microparticle on an Abbott ARCHITECT i2000SR Immunoassay Analyzer (Abbott Laboratories, Abbott Park, IL, USA) following the manufacturer’s instructions.

### 2.3 Data collection

We collected the patients’ demographic characteristics, clinical data, and laboratory findings from the medical records of the West China Second University Hospital of Sichuan University. They included the following parameters: complete blood cell count, lymphocyte subpopulations, biochemistry indexes, blood coagulation function, serum concentrations of immunoglobulins (IgG, IgA, and IgM), and complement. In addition, we also obtained their organ involvement and SLEDAI scores. Hematological involvement includes leukopenia, thrombocytopenia, and/or anemia. Cardiovascular involvement encompasses pericarditis, myocarditis, valvular disease and/or coronary artery disease. Pulmonary involvement includes manifestations affecting the pleura, diaphragm, parenchyma, and/or vasculature.

Some available renal biopsy reports were utilized to analyze the relationship between LN pathological classification and HE4 levels. According to the 2003 ISN/RPS classification, LN was categorized into six types: class I (minimal mesangial LN), class II (mesangial proliferative LN), class III (focal LN), class IV (diffuse segmental or global LN), class V (membranous LN), and class VI (advanced sclerosing LN) (27). Patients were further classified into proliferative LN (PLN), which included patients from classes III, IV, III+V, and IV+V, and non-PLN, comprising patients from classes I, II, V and VI.

### 2.4 Statistical analysis

Comparisons between groups were performed using the independent Student t-test, Mann–Whitney U test, Chi-square test, or Fisher’s exact test, as appropriate. Data were presented as mean ± standard deviation (SD) for parametric data and median (interquartile range, IQR) for non-parametric data. Categorical variables were expressed as numbers (%). Spearman’s correlation test was used to determine the associations between serum HE4 and other variables. The receiver operator characteristic (ROC) curve analysis was generated to evaluate sensitivity, specificity, Youden index (sensitivity + specificity − 1), and areas under the ROC curve (AUCs) with a 95% confidence interval (CI). All data analyses were calculated using SPSS version 22.0 statistical software (SPSS, Chicago, IL, USA) or GraphPad Prism 9 (GraphPad Software, San Diego, CA, USA). All statistical tests were two-sided, and *P* < 0.05 were considered statistically significant.

## 3 Results

### 3.1 Characteristics of patients with pSLE and HCs

**Table 1** shows the characteristics of the patients. We analyzed 137 patients with pSLE and 75 HCs. Sex and age were not significantly different between patients with the pSLE and HCs. Based on the SLEDAI score, the participants were divided into mild (n = 58), moderate (n=26), and severe disease activity groups (n = 53). The overall median SLEDAI score was 8 (4–16). Frequency statistics were conducted on 107 patients with complete clinical symptom records. The findings revealed that the most frequently affected systems were renal (85/107, 79.44%), hematological (47.66%, 47/107), musculoskeletal (25.23%, 27/107), and nervous systems (24.30%, 26/107). Moreover, the most common symptoms in pSLE were facial erythema (60.75%, 65/107) and oral ulcers (36.45%, 39/107). Based on age, the participants were subgrouped into three groups: prepubertal, ≤7 years (n = 12); peripubertal, 8–13 years (n = 94); and adolescent, 14–18 years (n = 31) (**Supplementary Table S1**). Prepubertal patients with pSLE had more common oral ulcers compared with older patients with pSLE (66.67%, 8/12 [prepubertal] vs. 30.85%, 29/94 [peripubertal] vs. 32.26%, 10/31 [adolescent]; *P* = 0.047). Other clinical features showed no statistically significant differences between groups (*P* > 0.05).

**Table 1:** Demographic and clinical characteristics of patients with pSLE and matched HCs.

	pSLE (n = 137)	HCs (n = 75)
Demography		
Sex (male,%)	14 (10.22%)	7(9.33%)
Age (mean ± SD)	11.72 ± 2.53	11.26 ± 2.68
SLEDAI score (median, IQR)	8 (4–16)	―
Clinical manifestations, n (%)		
Facial erythema	65 (60.75%)	―
Sun allergy	18 (16.82%)	―
Oral ulcers	39 (36.45%)	―
Hair loss	24 (22.43%)	―
Renal involvement	85 (79.44%)	―
Hematological involvement	47 (47.66%)	―
Musculoskeletal involvement	27 (25.23%)	―
Cardiovascular involvement	25 (23.36%)	―
Nervous system involvement	26 (24.30%)	―
Gastrointestinal involvement	20 (18.69%)	―
Pulmonary involvement	18 (16.82%)	―
Laboratory (mean ± SD)		
WBC count (10^9^/L)	7.84 ± 3.81	―
RBC count (10^12^/L)	3.97 ± 0.87	―
Lymphocyte count (10^9^/L)	1.79 ± 1.09	―
PLT count (10^9^/L)	248.4 ± 128.2	―
HGB (g/L)	115.4 ± 23.5	―
C3 (g/L)	0.69 ± 0.38	―
C4 (g/L)	0.14 ± 0.10	―
C1q (g/L)	18.37 ± 4.86	―
IgG (g/L)	11.46 ± 6.24	―
Ig A(g/L)	1.61 ± 0.90	―
IgM (g/L)	1.08 ± 0.62	―
IgE (g/L)	307.58 ± 548.67	―
ESR (mm/h)	20.24 ± 20.72	―
CRP (mg/L)	2.40 ± 7.84	―
ALT (g/L)	40.02 ± 91.79	―
AST (g/L)	49.28 ± 174.29	―
Tbil (umol/L)	9.77 ± 11.92	―
Dbil (umol/L)	3.55 ± 9.12	―
ALP (U/L)	113.19 ± 76.27	―
GGT (U/L)	38.01 ± 53.58	―
BUN (mmol/L)	7.05 ± 5.17	―
Cr (µmol/L)	54.08 ± 30.90	―
UA (µmol/L)	335.06 ± 116.85	―
TG (mmol/L)	2.27 ± 1.53	―
TC (mmol/L)	4.86 ± 1.73	―
LDL (mmol/L)	2.97 ± 1.54	―
HDL (mmol/L)	1.33 ± 0.69	―
PT (s)	11.07 ± 1.30	―
APTT (s)	26.82 ± 4.78	―
TT (s)	17.51 ± 1.46	―
Fbg (mg/dL)	277.49 ± 114.05	―
%CD3^+^T cells	77.21 ± 10.03	―
%CD3^+^CD4^+^T cells	29.98 ± 10.82	―
%CD3^+^CD8^+^T cells	42.14 ± 11.45	―
CD3^+^CD4^+^/CD3^+^CD8^+^	0.77 ± 0.43	―
%CD19^+^B cells	0.27 ± 0.25	―
%CD3^−^CD16^+^CD56^+^NK cells	6.26 ± 5.57	―

SD, standard deviation; IQR, interquartile range; WBC, white blood cell count; RBC, red blood cell count; PLT, platelet; HGB, hemoglobin; C3, complement 3; C4, complement 4; C1q, complement 1q; IgG, immunoglobulin G; IgA, immunoglobulin A; IgM, immunoglobulin M; IgE, immunoglobulin E; ESR, erythrocyte sedimentation rate; CRP, C-reactive protein; ALT, alanine aminotransferase; AST, aspartate aminotransferase; Tbil, total bilirubin; Dbil, direct bilirubin; ALP, alkaline phosphatase; GGT, gamma-glutamyl transpeptidase; BUN, blood urea nitrogen; Cr, creatinine; UA, uric acid; TG, triglyceride; TC, total cholesterol; LDL, low-density lipoprotein; HDL, high-density lipoprotein; PT, prothrombin time; APTT, activated partial thromboplastin time; TT, thrombin time; Fbg, fibrinogen; pSLE, pediatric-onset systemic lupus erythematosus; HCs, healthy controls.

### 3.2 Serum HE4 levels were significantly elevated in pSLE

**Figure 1:**
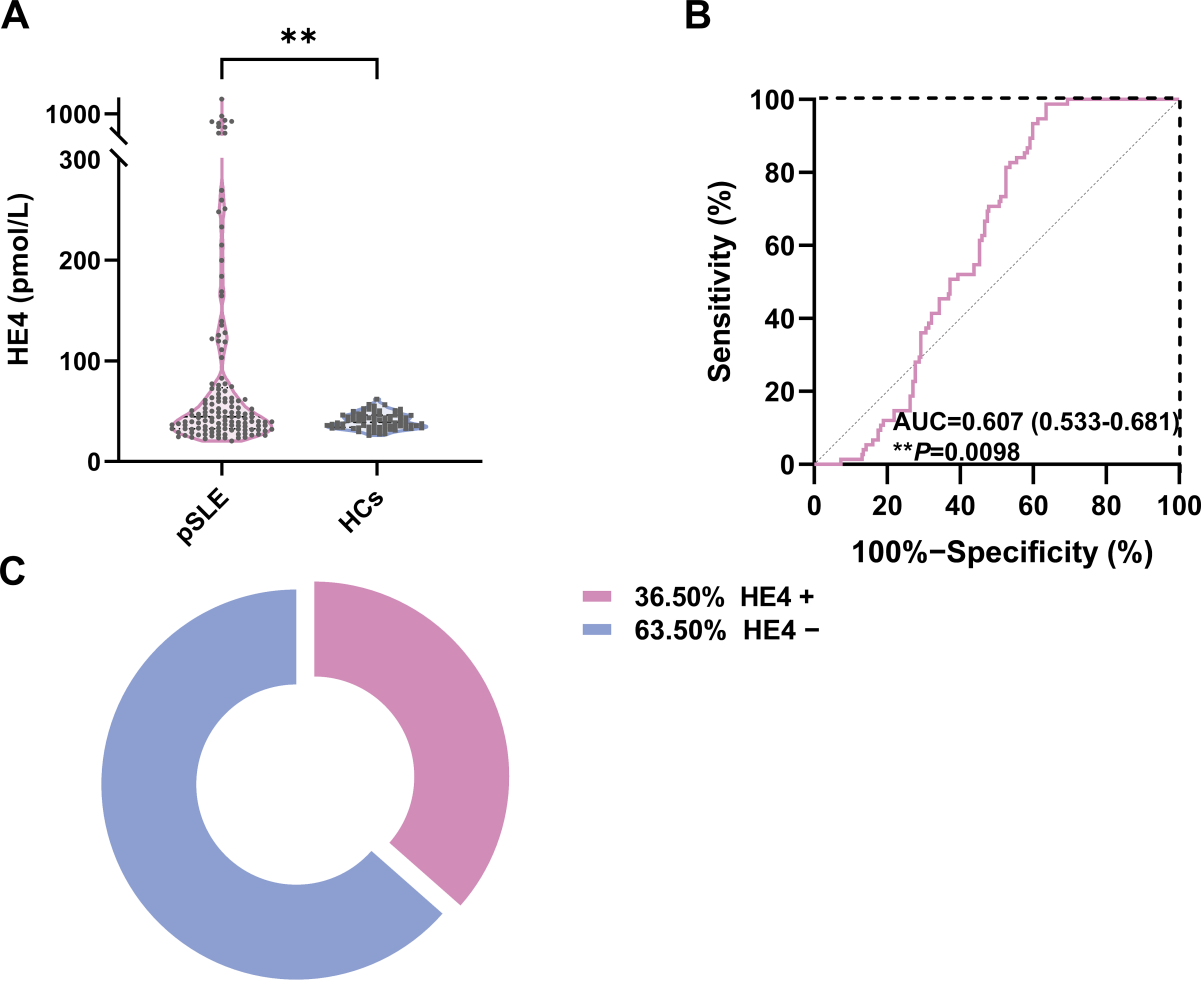
Serum HE4 levels in pSLE and HCs. **(A)** Violin plots showing the serum HE4 levels were significantly higher in the patients with pSLE than in HCs. **(B)** ROC curves for the HE4 cutoff value between pSLE and HCs. **(C)** The distribution of HE4-positive (HE4+) and HE4-negative (HE4−) pSLE based on the cutoff value. HE4, human epididymis protein 4; AUC, area under the curve; pSLE, pediatric-onset systemic lupus erythematosus; HCs, healthy controls. (**P < 0.01).

The serum HE4 levels in patients with pSLE (44.6 pmol/L; IQR, 32.5–73.5) were significantly higher than those in HCs (38.9 pmol/L; IQR, 34–46.1) (**Figure 1A**; *P* < 0.01). ROC analysis revealed that the cutoff value to distinguish pSLE from HCs was 56.9 pmol/L, with an AUC of 0.607 (95% CI, 0.533–0.681, *P* = 0.0098) (**Figure 1B**).

**Figure 2:**
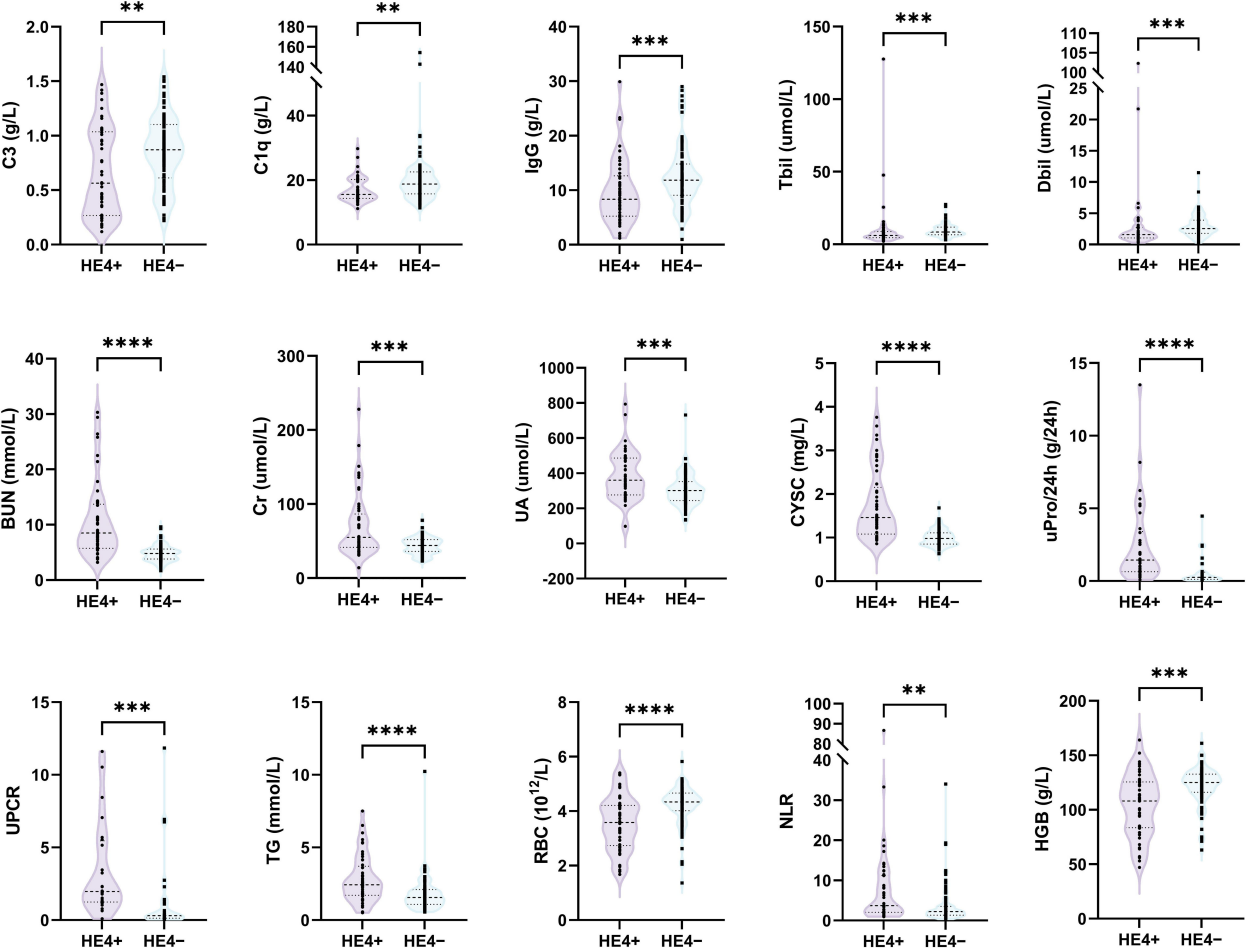
Comparison of laboratory test results between HE4-positive (HE4+) and HE4-negative (HE4–) pSLE. HE4, human epididymis protein 4; C3, complement 3; C1q, complement 1q; IgG, immunoglobulin G; Tbil, total bilirubin; Dbil, direct bilirubin; BUN, blood urea nitrogen; Cr, creatinine; UA, uric acid; CYSC, Cystatin C; uPro/24 h, urine protein/24 h; UPCR, urinary protein/creatinine ratio; TG, triglyceride; RBC, red blood cell count; NLR, neutrophil-to-lymphocyte ratio; PLT, platelet; HGB, hemoglobin. (**P < 0.01, ***P < 0.001, ****P < 0.0001).

Based on the cutoff value, patients with pSLE were divided into HE4 positive (HE4+, n = 50) and HE4 negative (HE4−, n = 87) (**Figure 1C**). The laboratory test results between these two groups were compared (**Figure 2**; **Supplementary Figure S1**, **Supplementary Table S2**). Compared with HE4− patients, HE4+ patients had shorter disease duration (*P* = 0.009, **Supplementary Table S2**). Multiple laboratory parameters (**Figure 2**, *P* < 0.01) and other laboratory test results (**Supplementary Figure S1**, *P* < 0.05) showed statistically significant differences between HE4+ and HE4− patients. Significantly increased parameters included neutrophil count, neutrophil-to-lymphocyte ratio (NLR), gamma-glutamyl transpeptidase (GGT), blood urea nitrogen (BUN), creatinine (Cr), uric acid (UA), cystatin C (CYSC), urine protein/24 h uPro/24 h), urinary protein/creatinine ratio (UPCR), triglyceride (TG), total cholesterol (TC). Parameters, such as red blood cell (RBC) count, lymphocyte count, platelet (PLT) count, hemoglobin (HGB), complement 3 (C3), complement 1q (C1q), immunoglobulin (Ig) G, IgE, total bilirubin (Tbil), and direct bilirubin (Dbil), significantly decreased.

### 3.3 Elevated HE4 levels were related to organ involvement in pSLE

**Figure 3:**
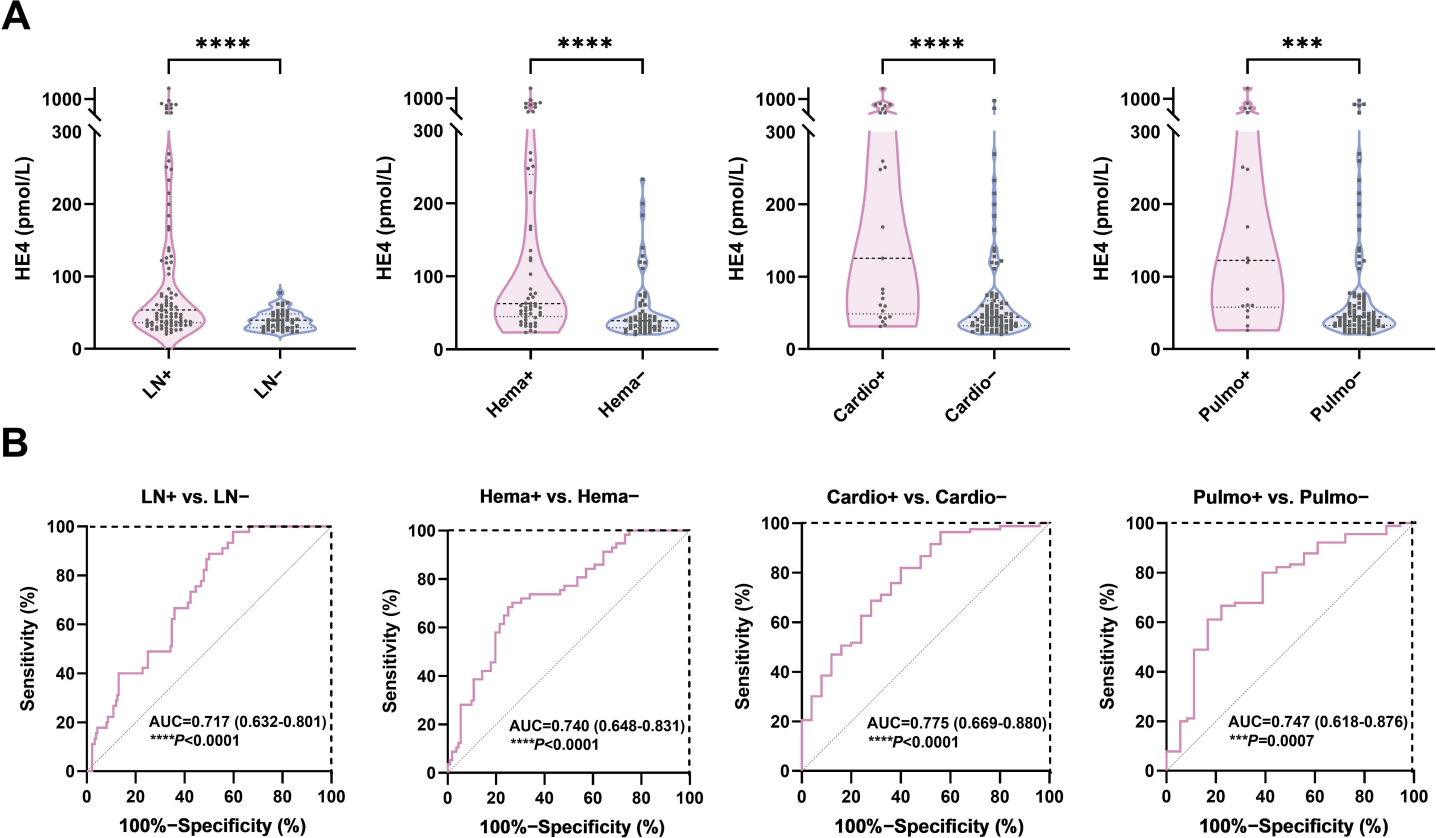
Diagnostic potential of HE4 in pSLE-related organ involvement. **(A)** Violin plots showing the serum HE4 levels were significantly higher in pSLE patients with organ involvement than those without. **(B)** ROC curve analyses on the clinical performance of HE4 in identifying patients with pSLE with and without organ involvement. HE4, human epididymis protein 4; AUC, area the under curve; LN, lupus nephritis; Hema, hematological involvement; Cardio, cardiovascular involvement; Pulmo, pulmonary involvement. (***P < 0.001, ****P < 0.0001).

Patients with specific organ involvement had higher HE4 levels than those without (**Figure 3A**). Serum HE4 levels were higher in pSLE patients with LN (LN+, n = 85; HE4, 57.1 pmol/L; IQR 35.25–137.4) than those without LN (LN−, n = 22; HE4, 40.2 pmol/L; IQR, 30.8–52.48) (*P* = 0.0086) (**Table 2**). In addition, several urine-related symptoms, such as proteinuria, hematuria, pyuria, and cylindruria, that can reflect renal involvement in patients with pSLE were also associated with higher HE4 levels. With respect to the LN pathological classification, **Supplementary Figure S2** indicates that there was no statistical difference in HE4 levels between the PLN and non-PLN groups (median, 60.0 pmol/L vs. 45.9 pmol/L, *P*=0.223).

**Table 2:** Comparison of HE4 levels between patients with or without corresponding clinical manifestations.

Clinical manifestations (median, IQR)	HE4 levels of patients with clinical manifestations (mol/L)	Number of patients with organ involvement	HE4 levels of patients without clinical manifestations (mol/L)	Number of patients without organ involvement	*P-*value
Facial erythema	47.6 (32.5–75.8)	65	55.85 (34.48–128.9)	42	0.381
Sun allergy	48.25 (39.53–130.7)	18	48.7 (32.8–79.95)	89	0.613
Oral ulcers	53.3 (35.9–111)	39	47.75 (32.5–119.4)	68	0.679
Hair loss	51.35 (36.33–123.7)	24	46.9 (33.1–118.8)	83	0.663
Renal involvement	57.1 (35.25–137.4)	85	40.2 (30.8–52.48)	22	**0.0086**
Hematological involvement	63.2 (46.9–251.2)	51	38.6 (28.4–61.53)	46	**<0.0001**
Musculoskeletal involvement	48.6 (35.9–74.5)	27	50.55 (32.65–127.3)	80	0.614
Cardiovascular involvement	125.5 (48.75–440.7)	25	43.9 (32.25–68.13)	81	**<0.0001**
Nervous system involvement	48.25 (32.45–188.5)	26	48.7 (35.25–114.9)	81	0.89
Gastrointestinal involvement	46.9 (32.1–120.7)	21	48.8 (34.48–113.0)	86	0.85
Pulmonary involvement	122.6 (57.83–390.3)	18	44.4 (32.5–73.45)	89	**0.0008**
ANA>1:320	39.7 (31.65–66.25)	73	48.7 (35.7–77.1)	63	0.111
Anti-histone	60.2 (38.35–170.3)	25	43.6 (32.1–63)	111	**0.031**
Anti-smith	39.7 (31.65–53.7)	17	44.7 (33.10–75.70)	119	0.2
Anti-RNP	39.7 (31.8–54.6)	47	48.6 (35.3–79.95)	89	0.077
Hypocomplementemia	53.05 (35.5–130.7)	66	39.9 (31.2–59.1)	71	**0.0056**
Proteinuria	75.7 (44.35–224.1)	61	36.6 (29–47.55)	76	**<0.0001**
Hematuria	119.6 (54.6–251.2)	39	39.2 (31.2–50.93)	98	**<0.0001**
Pyuria	71.8 (43.7–249.6)	17	43.3 (32.1–63.35)	120	**0.0087**
Cylindruria	168.6 (114.9–339.1)	25	39.65 (31.5–53.18)	112	**<0.0001**

Values presented in bold indicate that the associated *P*-value is less than 0.05.

Serum HE4 levels were higher in patients with pSLE with hematological involvement (Hema+, n = 51; HE4, 63.2 pmol/L; IQR, 46.9–251.2) than those without (Hema−, n = 46; HE4, 38.6 pmol/L; IQR, 28.4–61.53) (*P* < 0.0001). Serum HE4 levels were higher in patients with pSLE with cardiovascular involvement (Cardio+, n = 26; HE4, 125.5 pmol/L; IQR, 48.75–440.7) than those without (Cardio−, n = 81; HE4, 43.9 pmol/L, IQR, 32.25–68.13) (*P* < 0.0001). Serum HE4 levels were higher in patients with pSLE with pulmonary involvement (Pulmo+, n = 18; HE4, 122.6 pmol/L; IQR, 57.83–390.3) than those without (Pulmo−, n = 89; HE4, 44.4 pmol/L; IQR, 32.5–73.45) (*P* = 0.0007). Elevated HE4 levels can predict potential damage to internal organs in patients with pSLE.

### 3.4 Diagnostic potential of HE4 in pSLE-related organ involvement

**Figure 3B** shows the ROC analysis on the clinical performance of HE4 in identifying patients with corresponding organ involvement. The ROC analysis showed AUCs of 0.717 (95% CI 0.632–0.801), 0.740 (95% CI, 0.648–0.831), 0.775 (95% CI, 0.669–0.880), and 0.747 (95% CI 0.618–0.876) for distinguishing patients with pSLE with LN, hematological, cardiovascular, and pulmonary involvement from those without, respectively, with sensitivity and specificity of 50.0% and 88.9% (*P* < 0.0001), 73.2% and 70.2% (*P* < 0.0001), 60.0% and 80.7% (*P* < 0.0001), and 77.8% and 66.7% (*P* = 0.0007), respectively. Furthermore, a HE4 level exceeding 78 pmol/L also demonstrated a specificity of 100% for the diagnosis of LN.

In addition, the association between variables and pSLE-associated LN was further assessed using univariate and multivariate analyses (**Table 3**). Univariate analysis showed that several variables were significantly associated with LN in patients with pSLE, including low C3 levels, IgG, Cr, BUN, RBC, HGB and high HE4 levels. Based on the correlation results, HE4 shows strong correlations with RBC, HGB, Cr, and BUN. By assessing the goodness of fit in their respective univariate models, HE4 served as the most significant variable, demonstrating the R-Square value of 0.263 and the –2Log Likelihood of 144.838. Subsequently, C3, IgG, and HE4 were subjected to a multivariate analysis. The results indicated that low C3 levels (OR, 4.005; 95%CI, 1.492-11.97; P = 0.0083), serum IgG levels (OR, 0.877; 95%CI, 0.807-0.947; P = 0.0012) and high HE4 levels (OR, 5.034; 95%CI, 1.81-16.58; P = 0.0036) were significantly associated with LN in patients with pSLE.

**Table 3:** Univariable and multivariable analyses for risk factors of pSLE associated LN.

Variables	Univariable analysis		Multivariable analysis	
	*P-*value	OR (95% CI)	*P-*value	OR (95% CI)
Age (years)	0.361	1.068 (0.928–1.230)		
Sex	0.344	1.901 (0.558–8.739)		
Serum C3 ≤ 0.70 g/L	**0.0063**	3.117 (1.418–7.334)	**0.0083**	4.005(1.492-11.97)
Serum C4 ≤ 0.11 g/L	0.247	1.554 (0.744–3.337)		
IgG (g/L)	**0.0024**	0.905 (0.845–0.963)	**0.0012**	0.877(0.807-0.947)
IgA (g/L)	0.798	0.798 (0.543–1.168)		
IgM (g/L)	0.827	0.954 (0.623–1.519)		
Cr (µmol/L)	**0.0053**	1.041 (1.015–1.073)		
BUN (mmol/L)	**0.0011**	1.450 (1.191–1.858)		
WBC count (10^9^/L)	0.144	1.082 (0.979–1.212)		
RBC count (10^12^/L)	**0.0021**	0.426 (0.236–0.707)		
PLT count (10^9^/L)	0.703	0.999 (0.997–1.002)		
HGB (g/L)	**0.0029**	0.970 (0.950–0.988)		
Serum HE4>53.95 pmol/L	**<0.0001**	8.0 (3.131–24.8)	**0.0036**	5.034(1.81-16.58)
ANA>1:320	0.955	1.021 (0.497–2.091)		

Values presented in bold indicate that the associated *P*-value is less than 0.05. Cutoff values for C3, C4, and HE4 were established through ROC analysis to differentiate between LN and non-LN.

OR, odds ratio; CI, confidence interval; C3, complement 3; C4, complement 4; IgG, immunoglobulin G; IgA, immunoglobulin A; IgM, immunoglobulin M; IgE, immunoglobulin E; Cr, creatinine; BUN, blood urea nitrogen; WBC, white blood cell count; RBC, red blood cell count; PLT, platelet; HGB, hemoglobin; HE4, human epididymis protein 4; ANA, antinuclear antibody; pSLE, pediatric-onset systemic lupus erythematosus; LN, lupus nephritis.

### 3.5 Serum HE4 levels were related to the pSLE severity

**Figure 4:**
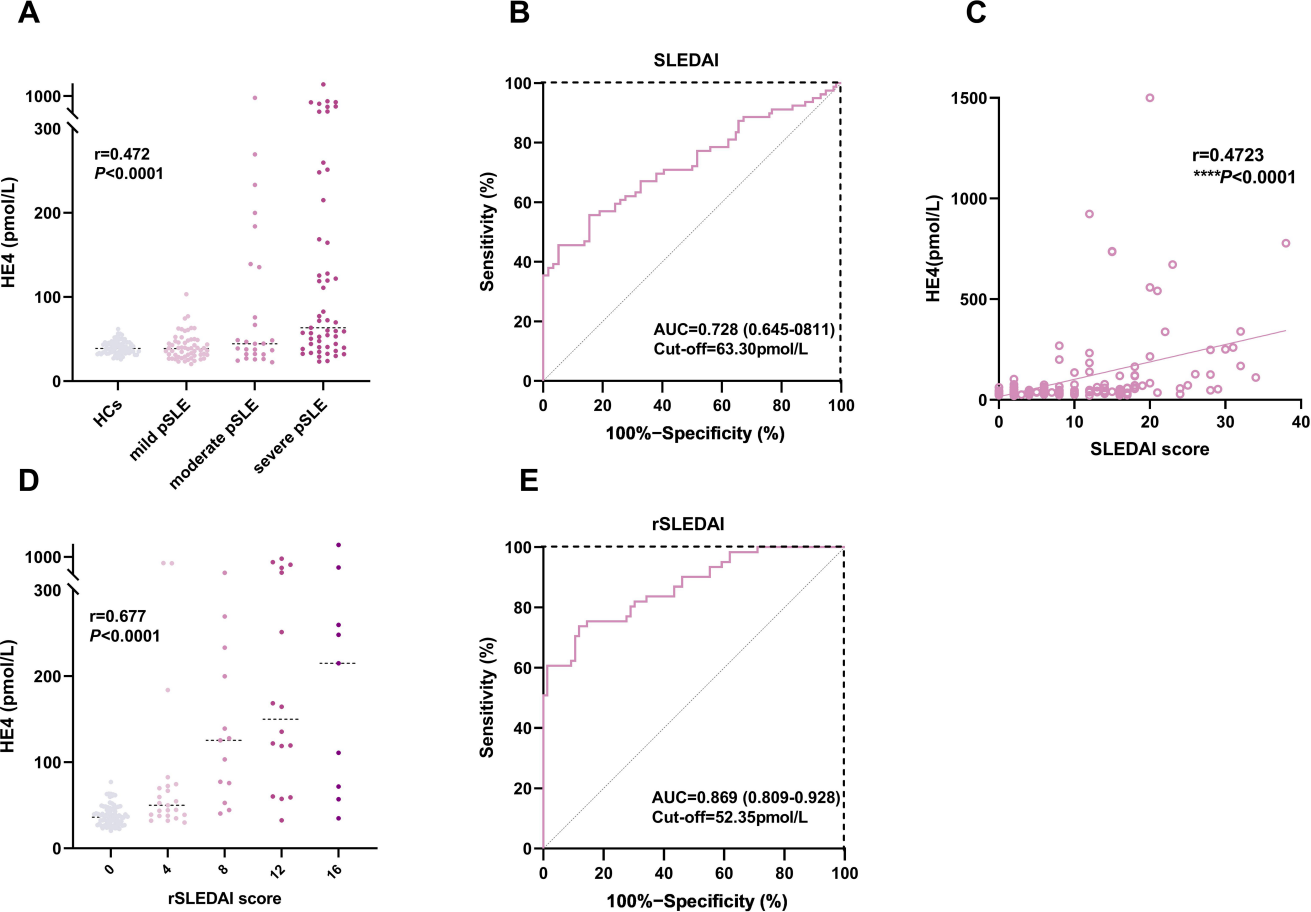
Associations between HE4 levels and disease activities. **(A)** HE4 levels in HCs and patients with pSLE with different SLEDAI scores. **(B)** ROC analysis in distinguishing patients with pSLE patients based on SLEDAI scores. **(C)** Correlation of HE4 levels and SLEDAI scores. **(D)** HE4 levels in patients with pSLE with different rSLEDAI scores. **(E)** ROC analysis in distinguishing patients with pSLE based on rSLEDAI scores. HE4, human epididymis protein 4; AUC, area the under curve; SLEDAI, systemic lupus erythematosus disease activity index; rSLEDAI, renal domains of SLEDAI; pSLE, pediatric-onset systemic lupus erythematosus. (****P < 0.0001).

We determined the serum HE4 levels in pSLE patients with different disease activities (**Figure 4**). **Figure 4A** shows that HE4 levels were significantly higher in moderate to severe disease activities (57.4 pmol/L, IQR, 37.7–164.5) compared with those with mild disease activities (38.8 pmol/L, IQR, 30.1–48.5) or HCs (38.9 pmol/L, IQR, 34.0–46.1) (*P* < 0.0001). The ROC analysis showed an AUC of 0.728 (95% CI, 0.645–0.811) for distinguishing patients with pSLE with moderate to severe disease activities from mild disease activity, with an optimal cutoff value of 63.30 pmol/L (*P* < 0.0001) (**Figure 4B**). A positive correlation (r = 0.4723, *P* < 0.0001) was observed between HE4 levels and SLEDAI scores (**Figure 4C**).

**Figure 4D** shows that HE4 levels were significantly higher in active renal disease activities (77.2 pmol/L, IQR, 47.4–224.1) than in inactive renal disease activities (36.1 pmol/L, IQR, 27.8–46.7) (*P* < 0.0001). The ROC analysis showed an AUC of 0.869 (95% CI, 0.809–0.928) for distinguishing moderate to active LN from inactive LN, with an optimal cutoff value of 52.35 pmol/L (*P* < 0.0001) (**Figure 4E**).

### 3.6 Correlations of HE4 with other laboratory parameters

**Figure 5:**
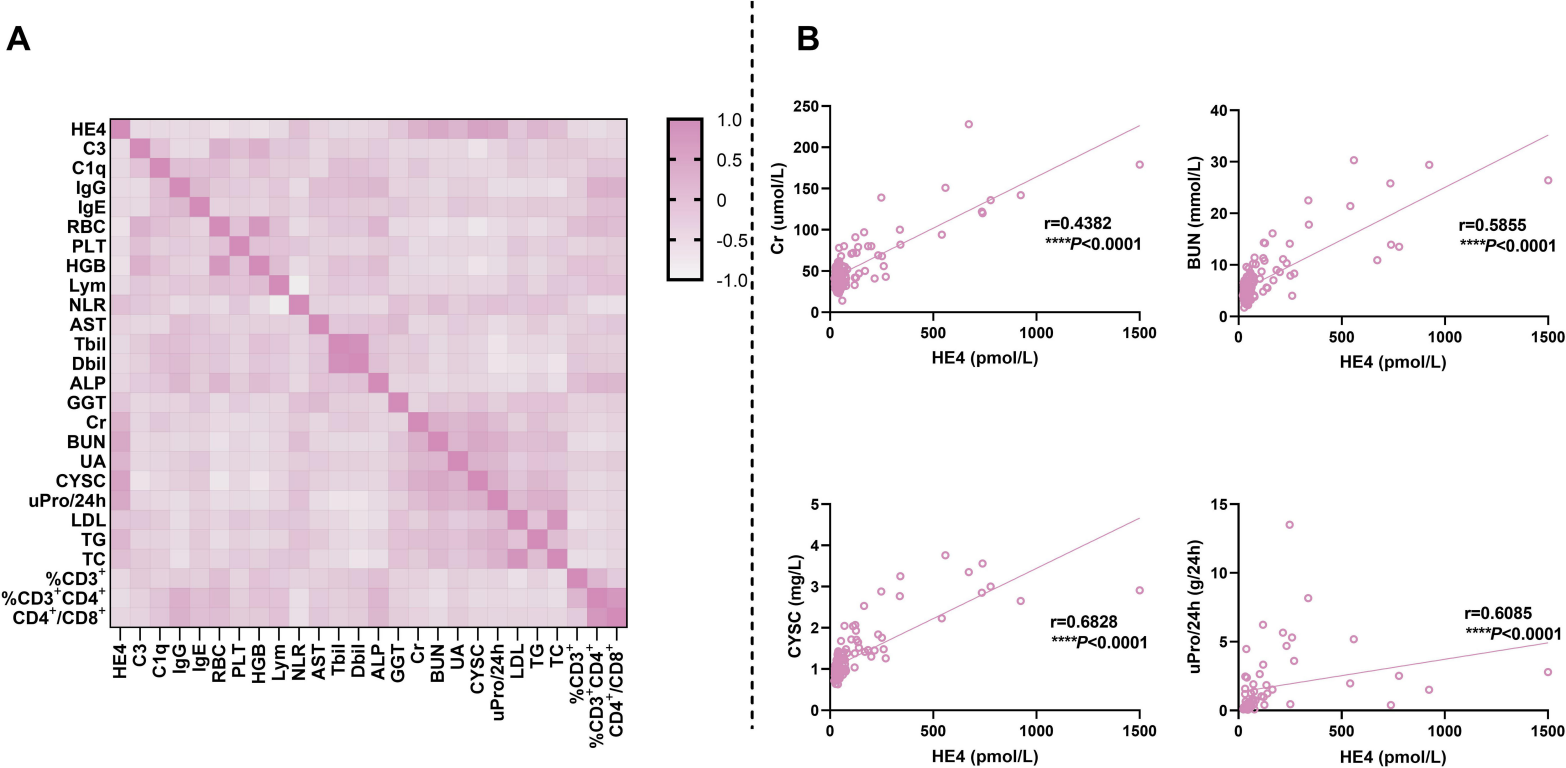
Correlations of serum HE4 levels with laboratory parameters. **(A)** Heatmap for the correlation between HE4 levels and other laboratory parameters. **(B)** Scatter plots for the correlation between HE4 levels and typical laboratory parameters. HE4, human epididymis protein 4; C3, complement 3; C1q, complement 1q; IgG, immunoglobulin G; IgE, immunoglobulin E; RBC, red blood cell; PLT, platelet; HGB, hemoglobin; Lym, lymphocyte; NLR, neutrophil-to-lymphocyte ratio; AST, aspartate aminotransferase; Tbil, total bilirubin; Dbil, direct bilirubin; ALP, alkaline phosphatase; GGT, gamma-glutamyl transpeptidase; Cr, creatinine; BUN, blood urea nitrogen; UA, uric acid; CYSC, cystatin C; uPro/24 h, urine protein/24 h; LDL, low-density lipoprotein; TG, triglyceride; TC, total cholesterol. (****P < 0.0001).

**Figure 5** and **Supplementary Table S3** show the correlations of serum HE4 levels with laboratory parameters in patients with pSLE. Serum HE4 levels were significantly positively associated with NLR (r = 0.228, *P* = 0.008), Cr (r = 0.438, *P* < 0.001), BUN (r = 0.586, *P* < 0.001), UA (r = 0.422, *P* < 0.001), CYSC (r = 0.683, *P* < 0.001), uPro/24 h (r = 0.609, *P* < 0.001), TG (r = 0.390, *P* < 0.001), and TC (r = 0.243, *P* = 0.01). However, serum HE4 levels were negatively correlated with C3 (r = −0.260, *P* = 0.002), C1q (r = −0.228, *P* = 0.008), IgG (r = −0.252, *P* = 0.003), IgE (r = −0.190, *P* = 0.027), RBC count (r = −0.432, *P* < 0.001), PLT count (r = −0.260, *P* = 0.003), HGB (r = −0.405, *P* < 0.001), lymphocyte count (r = −0.240, *P* = 0.005), Tbil (r = −0.215, *P* = 0.015), Dbil (r = −0.204, *P* = 0.022) and %CD3^+^CD4^+^T cells (r = −0.303, *P* = 0.003). **Figure 5B** presents several representative scatter plots of correlation. Several indicators showed a significant correlation between renal morbidity and HE4, such as Cr, BUN, UA, CYSC, uPro/24 h, etc.

## 4 Discussion

Although several studies have shown that patients with adult-onset SLE exhibit significantly elevated serum HE4 levels, as well as in other rheumatic diseases compared to HCs, research on the correlation between HE4 levels and disease activity in pSLE remains limited.

Our study showed higher HE4 levels among patients with pSLE with moderate to severe disease activity compared with those with mild pSLE and HCs. Moreover, HE4 levels were positively correlated with SLEDAI scores, further highlighting the role of HE4 in reflecting overall disease activity. Our findings are consistent with recent studies, indicating that HE4 consistently showed a strong correlation with LN (24). To assess its relationship further, we calculated the rSLEDAI score. The results showed that the correlation between HE4 and rSLEDAI was significantly stronger compared with SLEDAI, and the AUC performance was also superior. Furthermore, we found that high levels of HE4 were associated with the presence of LN in patients with pSLE in a multivariate logistic regression analysis. The positive associations with parameters indicative of renal involvement and inflammation, such as NLR, Cr, BUN, UA, CYSC, and uPro/24 h, indicated that HE4 may be involved in the inflammatory processes and renal pathologies in pSLE.

HE4 is a member of the whey acidic protein family and is widely expressed in the respiratory tract, nasopharynx, salivary gland, kidney, and other organs (10, 28). HE4 can suppress serine protease and matrix metalloproteinase activities and specifically inhibit their capacity to degrade type I collagen, thereby promoting the development of kidney fibrosis (29). A previous study reported that HE4 is a fibroblast-derived mediator of renal fibrosis (30). Ren et al. reported that renal dysfunction may lead to reduced HE4 clearance, resulting in elevated serum HE4 levels in patients with LN patients (22). In addition, there have been reports of HE4 expression in the kidney (31), and elevated levels of HE4 have been observed in patients with renal impairment (32). In this study, it was found that among patients with pSLE, a substantial proportion experienced kidney function impairment, accounting for 79.44%. Hence, this could potentially explain the increased HE4 levels in patients with pSLE as well. While the correlation between HE4 and renal involvement in pSLE is evident, no distinctions have been identified within specific renal pathological classifications. And the lack of statistical difference in HE4 levels between the PLN and non-PLN groups in pSLE is consistent with previous research, which indicates differences in adult LN but not in pediatric LN (24).

Serum HE4 levels were significantly elevated in patients with pSLE than in HCs, and the derived cutoff value of 56.9 pmol/L for distinguishing pSLE from HCs underscores the diagnostic potential of HE4. Our results were consistent with previous studies in adult patients with SLE patients (22, 33). The association of higher HE4 levels with shorter disease duration in HE4+ patients further support the use of HE4 as a biomarker for early disease detection. The significantly elevated plasma levels of HE4 in patients with pSLE were closely associated with various baseline laboratory parameters, indicating a close linkage between HE4 and the pathogenesis of pSLE. When patients with pSLE were classified as HE4+ or HE4− based on the cutoff values determined by ROC analysis, HE4+ patients showed higher baseline laboratory parameters (neutrophil count, NLR) and organ function indicators (Cr, BUN, UA, CYSC, GGT, uPro/24h, UPCR, TG, TC), suggesting that plasma HE4 levels can be utilized to predict potential organ damage in pSLE. These findings were further supported by the results of correlation analyses.

Our analysis extends the diagnostic potential of HE4 to specific organ involvement. HE4 levels were consistently higher in patients with organ involvement, particularly LN, hematological, cardiovascular, and pulmonary involvement. The correlation between HE4 and hematological parameters could potentially indicate an association with the blood system. HE4 may have multifaceted impacts on hematopoietic systems, particularly in the context of pSLE. Notably, existing literature does not currently report specific research on this topic, which could be a valuable area for future exploration. Many studies have reported the correlation between HE4 and cardiovascular and pulmonary system involvement. Yamamot et al. reported that HE4 predicted progressive fibrosis and cardiovascular events in patients with dilated cardiomyopathy (34). Tang et al. reported that HE4 is an independent predictor of cardiovascular death and heart failure-related rehospitalization in patients with ischemic cardiomyopathy (35). Lin et al. reported that HE4 is a new diagnostic biomarker for RA-associated interstitial lung disease (ILD) (36). Chen et al. reported that patients with pSS with pulmonary involvement had significantly higher HE4 levels than those without (17). Sun et al. reported that the percentage of IIM-related ILD in the HE4+ group was nearly twice as much as that in the HE4− group (18). The proportion of internal organ involvement was higher in HE4+ IgG4-RD patients (19), which was consistent with our findings showing that HE4 was significantly associated with specific organ involvement in patients with pSLE. These findings were supported by the ROC analysis, which showed moderate to high AUC values for HE4 in identifying pSLE with organ involvement, indicating HE4 as a promising biomarker for organ-specific disease activity.

Our study has several limitations. First, it was not a prospective study but rather reflected a real-world experience with routine clinical tests on HE4. Second, it was a single-center study, and the sample size was small, thereby predisposing to selection bias. Third, the number of kidney specimens in the histopathologic examination was rather small. Fourth, HE4 levels were only detected at baseline, and changes in HE4 levels after treatment are unknown. Further multicenter prospective studies with a larger cohort will enlighten the clinical use of HE4 in the clinical stratification of pSLE and other rheumatoid diseases.

In conclusion, our study showed that serum HE4 levels were significantly elevated in pSLE and associated with disease activity, organ involvement, and various laboratory parameters, indicating that HE4 could be a valuable biomarker for pSLE diagnosis and monitoring. However, further studies are needed to elucidate the underlying mechanisms of HE4 elevation in pSLE and validate its clinical utility in larger cohorts and diverse populations.

## Data Availability

The original contributions presented in the study are included in the article/**Supplementary Material**. Further inquiries can be directed to the corresponding authors.

## Glossary

ALP: alkaline phosphatase
IgM: immunoglobulin M
ALT: alanine aminotransferase
IIM: idiopathic inflammatory myopathies
ANA: antinuclear antibody
ILD: interstitial lung disease
APTT: activated partial thromboplastin time
IQR: interquartile range
AST: aspartate aminotransferase
LDL: low-density lipoprotein
AUC: area the under curve
LN: lupus nephritis
BUN: blood urea nitrogen
Lym: lymphocyte
C1q: complement 1q
NLR: neutrophil-to-lymphocyte ratio
C3: complement 3
PLN: proliferative lupus nephritis
C4: complement 4
PLT: platelet
CI: confidence interval
pSLE: pediatric-onset systemic lupus erythematosus
Cr: creatinine
pSS: primary Sjögren syndrome
CRP: C-reactive protein
PT: prothrombintime
CYSC: cystatin C
RA: rheumatoid arthritis
Dbil: direct bilirubin
RBC: red blood cell count
ESR: erythrocyte sedimentation rate
ROC: receiver operator characteristic
Fbg: fibrinogen
rSLEDAI: renal systemic lupus erythematosus disease activity index
GGT: gamma-glutamyl transpeptidase
SD: standard deviation
HCs: healthy controls
SLEDAI: systemic lupus erythematosus disease activity index
HDL: high-density lipoprotein
Tbil: total bilirubin
HE4: human epididymis protein 4
TC: total cholesterol
HGB: hemoglobin
TG: triglyceride
HPF: high-power field
TT: thrombin time
IgA: immunoglobulin A
UA: uric acid
IgE: immunoglobulin E
UPCR: urinary protein/creatinine ratio
IgG: immunoglobulin G
uPro/24 h: urine protein/24 h
IgG4-RD: IgG4-related disease
WBC: white blood cell count
