# Supplementary material for: Increased serum human epididymis protein 4 is associated with disease activity and systemic involvement in pediatric-onset systemic lupus erythematosus
Source: Front Immunol. 2024 Sep 10;15:1461987. doi: 10.3389/fimmu.2024.1461987 (PMC11419971; doi:10.3389/fimmu.2024.1461987)
Supplement: Supplementary file 1 [file DataSheet1.docx]

Table S1. Clinical features of pSLE subgroups based on their age.

|  | **Pre-pubertal subgroup (n=12)** | **Peri-pubertal subgroup (n=94)** | **Adolescence (n=31)** | ***P***-**value** |
| --- | --- | --- | --- | --- |
| **Demography** |  |  |  |  |
| Sex (male, %) | 1 (8.33%) | 12 (12.77%) | 1 (3.23%) | 0.307 |
| SLEDAI score (mean ± SD) | 13.83 ± 8.28 | 11.36 ± 9.06 | 8.29 ± 7.32 | 0.099 |
| rSLEDAI score (mean ± SD) | 4.00 ± 6.32 | 4.21 ± 5.30 | 2.84 ± 4.33 | 0.392 |
| **Clinical manifestations, n (%)** | |  |  |  |
| Facial erythema | 8 (66.67%) | 54 (57.45%) | 19 (61.29%) | 0.798 |
| Sun allergy | 1 (8.33%) | 14 (14.89%) | 8 (25.81%) | 0.264 |
| Oral ulcers | 8 (66.67%) | 29 (30.85%) | 10 (32.26%) | **0.047** |
| Hair loss | 2 (16.67%) | 18 (19.15%) | 9 (29.03%) | 0.467 |
| Renal involvement | 6 (50.00%) | 63 (67.02%) | 23 (74.19%) | 0.317 |
| Hematological involvement | 7 (58.33%) | 39 (41.49%) | 10 (32.26%) | 0.289 |
| Musculoskeletal involvement | 4 (33.33%) | 24 (25.53%) | 7 (22.58%) | 0.769 |
| Cardiovascular involvement | 1 (8.33%) | 20 (21.28%) | 4 (12.90%) | 0.375 |
| Nervous system involvement | 4 (33.33%) | 21 (22.34%) | 3 (9.68%) | 0.162 |
| Gastrointestinal involvement | 3 (25.00%) | 16 (17.02%) | 2 (6.45%) | 0.228 |
| Pulmonary involvement | 2 (16.67%) | 14 (14.89%) | 2 (6.45%) | 0.45 |

Values presented in bold indicate that the associated *P*-value is less than 0.05.

SD, standard deviation; SLEDAI, systemic lupus erythematosus disease activity index; rSLEDAI, renal domains of SLEDAI; pSLE, pediatric-onset systemic lupus erythematosus.

[pre-pubertal (≤7 years), peri-pubertal (8–13 years) or adolescent (14–18 years)].

Table S2. Characteristics of patients with pSLE according to HE4 status.

|  | **HE4 positive(n=50)** | **HE4 negative(n=87)** | ***P* value** |
| --- | --- | --- | --- |
| **Demography** |  |  |  |
| Sex (male,%) | 6, 12.0% | 8, 9.20% | 0.77 |
| Age (mean ± SD) | 11.51 ± 2.81 | 11.92 ± 2.36 | 0.167 |
| Age at onset (years, mean ± SD) | 10.21 ± 2.56 | 10.48 ± 2.21 | 0.404 |
| Disease duration (months, median, IQR) | 357.77 ± 547.00 | 474.49 ± 518.14 | **0.009** |
| **Laboratory (median, IQR)** |  |  |  |
| WBC count (10^9^/L) | 7.6 (5.5-11.25) | 6.55 (5.03-8.93) | 0.106 |
| RBC count (10^12^/L) | 3.58 (2.74-4.21) | 4.34 (4.02-4.66) | **<0.0001** |
| Neutrophil count (10^9^/L) | 5.63 (3.5-8.1) | 4.02 (2.53-6.27) | **0.011** |
| Lymphocyte count (10^9^/L) | 1.46 (0.80-2.02) | 1.65 (1.07-2.67) | **0.017** |
| NLR | 3.71 (2.06-11.26) | 2.25 (1.33-3.54) | **0.001** |
| PLT count (10^9^/L) | 201 (137-281) | 241 (192.3-300) | **0.019** |
| HGB (g/L) | 108 (83.5-125.5) | 125 (116-132.8) | **0.0002** |
| C3 (g/L) | 0.57 (0.27-1.04) | 0.87 (0.61-1.10) | **0.0026** |
| C4 (g/L) | 0.095 (0.06-0.23) | 0.16 (0.09-0.22) | 0.071 |
| C1q (g/L) | 15.58 (14.33-20.18) | 18.79 (15.72-22.53) | **0.0017** |
| IgG (g/L) | 8.35 (5.25-12.65) | 11.85 (9.09-14.80) | **0.0009** |
| IgA (g/L) | 1.58 (0.83-2.28) | 1.63 (1.03-2.26) | 0.316 |
| IgM (g/L) | 0.78 (0.47-1.23) | 0.95 (0.67-1.29) | 0.129 |
| IgE (g/L) | 43.95 (18.40-151.8) | 128.0 (18.85-339.3) | **0.031** |
| ESR (mm/h) | 10 (2-37) | 12.5 (3-33.75) | 0.957 |
| CRP (mg/L) | 0.50 (0.50-3.60) | 0.50 (0.50-1.70) | 0.592 |
| ALT (U/L) | 23 (13.5-42.5) | 23.5 (15.75-40.25) | 0.877 |
| AST (U/L) | 23 (19-31) | 25.5 (20.75-32) | 0.397 |
| Tbil (umol/L) | 6 (4.6-8.85) | 8.45 (6.58-11.80) | **0.001** |
| Dbil (umol/L) | 1.6 (1.05-2.75) | 2.55 (1.78-3.90) | **0.0008** |
| ALP (U/L) | 77 (55-155) | 85 (65.5-131.5) | 0.293 |
| GGT (U/L) | 26 (17-48) | 19.5 (13-36.25) | **0.043** |
| BUN (mmol/L) | 8.5 (5.75-13.7) | 4.8 (3.8-5.6) | **<0.0001** |
| Cr (umol/L) | 55 (41.5-86.5) | 44 (36-52) | **0.0001** |
| UA (umol/L) | 360 (276-486) | 301 (244-352) | **0.0003** |
| CYSC (mg/L) | 1.46 (1.08-2.15) | 0.98 (0.85-1.11) | **<0.0001** |
| uPro/24h (g/24h) | 1.44 (0.64-3.40) | 0.25 (0.12-0.62) | **<0.0001** |
| UPCR | 1.97 (1.24-5.45) | 0.31 (0.13-1.29) | **0.0002** |
| TG (mmol/L) | 2.43 (1.70-3.71) | 1.56 (1.09-2.11) | **<0.0001** |
| TC (mmol/L) | 4.91 (3.72-6.69) | 3.97 (3.49-5.24) | **0.036** |
| LDL (mmol/L) | 2.8 (1.9-4) | 2.5 (1.95-3.15) | 0.323 |
| HDL (mmol/L) | 1.16 (0.82-1.64) | 1.18 (0.89-1.53) | 0.959 |
| PT (s) | 10.7 (10.3-11.43) | 10.8 (10.2-11.65) | 0.824 |
| APTT (s) | 25.1 (23.5-28.28) | 26.2 (23.55-29.2) | 0.483 |
| TT (s) | 17.3 (16.3-18.83) | 17.2 (16.6-18.05) | 0.747 |
| Fbg (mg/dL) | 231.5 (180.8-372.8) | 268 (218-288.5) | 0.601 |
| INR | 1.00 (0.94-1.05) | 1.02 (0.93-1.07) | 0.772 |
| %CD3^+^T cells | 78.5 (69.03-84.83) | 78.8 (73.55-86.5) | 0.188 |
| %CD3^+^CD4^+^T cells | 26.95 (18.03-31.93) | 32.4 (26.15-40.2) | **0.0017** |
| %CD3^+^CD8^+^T cells | 41.8 (37.28-52.03) | 40.9 (30.8-48.35) | 0.227 |
| CD3^+^CD4^+^/CD3^+^CD8^+^ | 0.6 (0.4-0.83) | 0.8 (0.5-1.15) | **0.022** |
| %CD19^+^B cells | 13.8 (6.5-24) | 11 (6.45-19.85) | 0.281 |
| %CD16^+^CD56^+^NK cells | 4.35 (2.25-8.7) | 3.9 (2.2-8.15) | 0.989 |

SD, standard deviation; IQR, interquartile range; WBC, white blood cell; RBC, red blood cell; NLR, neutrophil-to-lymphocyte ratio; PLT, platelet; HGB, hemoglobin; C3, complement 3; C4, complement 4; C1q, complement 1q; IgG, immunoglobulin G; IgA, immunoglobulin A; IgM, immunoglobulin M; IgE, immunoglobulin E; ESR, erythrocyte sedimentation rate; CRP, C-reactive protein; ALT, alanine aminotransferase; AST, aspartate aminotransferase; Tbil, total bilirubin; Dbil, direct bilirubin; ALP, alkaline phosphatase; GGT, gamma-glutamyl transpeptidase; BUN, blood urea nitrogen; Cr, creatinine; UA, uric acid; CYSC, Cystatin C; uPro/24h, urine protein/24 hours; UPCR, urinary protein/creatinine ratio; TG, triglyceride; TC, total cholesterol; LDL, low density lipoprotein; HDL, high density lipoprotein; PT, prothrombin time; APTT, activated partial thromboplastin time; TT, thrombin time; Fbg, fibrinogen; INR, international normalized ratio; HE4, human epididymis protein 4; pSLE, pediatric-onset systemic lupus erythematosus; HCs, healthy controls.

Table S3. The correlations of serum HE4 levels with laboratory parameters in patients with pSLE.

|  | **r** | **95%CI** | ***P-*value** |
| --- | --- | --- | --- |
| C3 | -0.260 | -0.4150 to -0.09139 | 0.002 |
| C1q | -0.228 | -0.3859 to -0.05702 | 0.008 |
| IgG | -0.252 | -0.4075 to -0.08250 | 0.003 |
| IgE | -0.190 | -0.3514 to -0.01713 | 0.027 |
| RBC | -0.432 | -0.5649 to -0.2784 | 2.01E-07 |
| PLT | -0.260 | -0.4160 to -0.08867 | 0.003 |
| HGB | -0.405 | -0.5413 to -0.2468 | 1.37E-06 |
| Lym | -0.240 | -0.3983 to -0.06761 | 0.005 |
| NLR | 0.228 | 0.05493 to 0.3876 | 0.008 |
| AST | -0.102 | -0.2766 to 0.07831 | 0.252 |
| Tbil | -0.215 | -0.3794 to -0.03688 | 0.015 |
| Dbil | -0.204 | -0.3693 to -0.02517 | 0.022 |
| ALP | -0.165 | -0.3347 to 0.01574 | 0.065 |
| GGT | 0.133 | -0.04741 to 0.3050 | 0.136 |
| Cr | 0.438 | 0.2817 to 0.5720 | 2.3E-07 |
| BUN | 0.586 | 0.4545 to 0.6918 | 3.89E-13 |
| UA | 0.422 | 0.2636 to 0.5587 | 6.89E-07 |
| CYSC | 0.683 | 0.5743 to 0.7678 | 6.84E-19 |
| uPro/24h | 0.609 | 0.4091 to 0.7525 | 4.03E-07 |
| LDL | 0.126 | -0.06734 to 0.3103 | 0.187 |
| TG | 0.390 | 0.2140 to 0.5411 | 2.36E-05 |
| TC | 0.243 | 0.05426 to 0.4158 | 0.010 |
| %CD3^+^T cells | -0.190 | -0.3866 to 0.02241 | 0.071 |
| %CD3^+^CD4^+^T cells | -0.303 | -0.4839 to -0.09755 | 0.003 |
| CD3^+^CD4^+^/CD3^+^CD8^+^ | -0.183 | -0.3804 to 0.02968 | 0.082 |
| Anti-dsDNA antibody | 0.134 | -0.04008 to 0.3003 | 0.1197 |

CI, confidence interval ; C3, complement 3; C1q, complement 1q; IgG, immunoglobulin G; IgE, immunoglobulin E; RBC, red blood cell; PLT, platelet; HGB, hemoglobin; Lym, lymphocyte; NLR, neutrophil-to-lymphocyte ratio; AST, aspartate aminotransferase; Tbil, total bilirubin; Dbil, direct bilirubin; ALP, alkaline phosphatase; GGT, gamma-glutamyl transpeptidase; BUN, blood urea nitrogen; Cr, creatinine; UA, uric acid; CYSC, Cystatin C; uPro/24h, urine protein/24 hours; LDL, low density lipoprotein; TG, triglyceride; TC, total cholesterol; HE4, human epididymis protein 4; pSLE, pediatric-onset systemic lupus erythematosus.


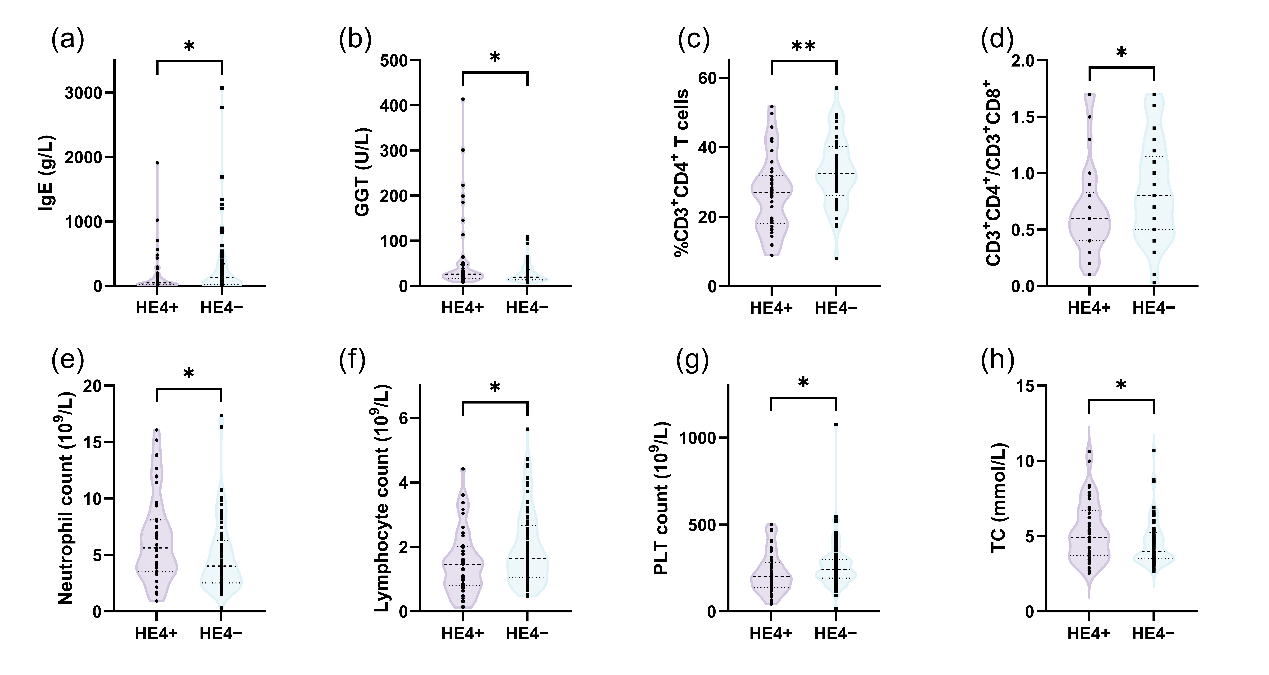


Figure S1. Comparison of the laboratory test results between patients with HE4-positive (HE4+) and HE4-negative (HE4–) pSLE.

IgE, immunoglobulin E; GGT, gamma-glutamyl transpeptidase; PLT, platelet; TC, total cholesterol; HE4, human epididymis protein 4; pSLE, pediatric-onset systemic lupus erythematosus.


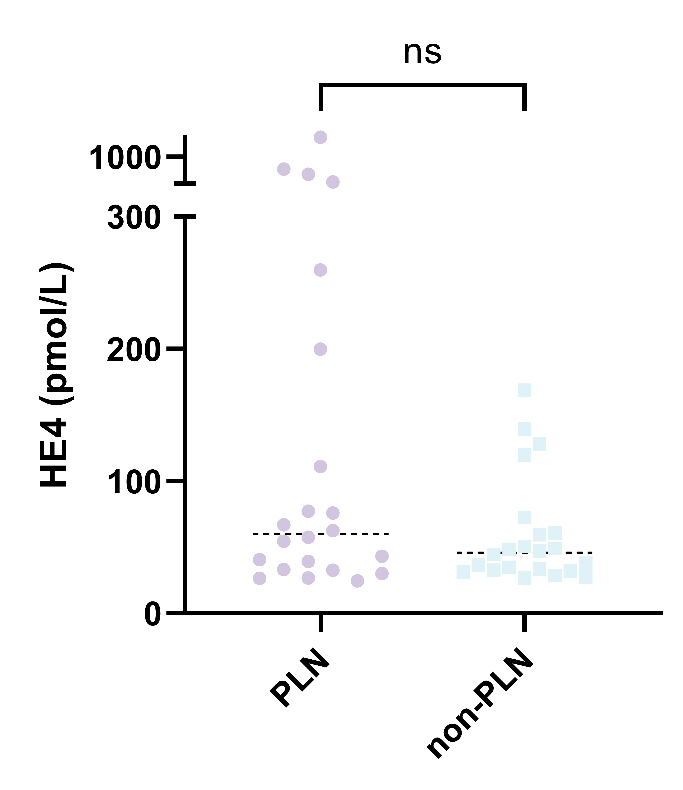


Figure S2. Comparison of serum HE4 levels between the PLN and non-PLN patients.

HE4, human epididymis protein 4; PLN, proliferative lupus nephritis.
